# Spontaneous aneurysm of a common digital artery: a rare entity

**DOI:** 10.1590/1677-5449.202400832

**Published:** 2025-02-14

**Authors:** Thilina Gunawardena

**Affiliations:** 1 Teaching Hospital, Department of Vascular and Transplant Surgery, Ratnapura, Sri Lanka.

**Keywords:** aneurysm, common digital artery, hand aneurysm, aneurisma, artéria digital comum, aneurisma da mão

## Abstract

Aneurysms of arteries of the hand are rarely encountered. Literature reports such aneurysms in the distal ulnar artery, the palmar artery, and the digital arteries. Most are false aneurysms involving trauma-induced defects of the vessel wall. True aneurysms with intact intimal, medial, and adventitial layers of the arterial wall are more unusual. In this case report, we present a 77-year-old previously healthy lady who was diagnosed with a true aneurysm of a common digital artery when she presented with a pulsatile lump over her left palm.

## Resumo

Aneurismas das artérias da mão são raros. A literatura disponível relata tais aneurismas na artéria ulnar distal, nas artérias palmares e nas artérias digitais. No entanto, a maioria consiste em pseudoaneurismas resultantes de trauma na parede do vaso. Aneurismas verdadeiros, com as camadas íntima, média e adventícia da parede arterial intactas, são ainda mais raros. Neste relato de caso, apresentamos uma mulher de 77 anos, previamente saudável, diagnosticada com aneurisma verdadeiro de artéria digital comum, após apresentar um nódulo pulsátil em sua palma esquerda.

## INTRODUCTION

Arteries of the hand can be infrequently affected by both true and false aneurysms. False aneurysms are a result of acute penetrating or blunt trauma. True aneurysms seem to be much rarer. The majority of such true aneurysms are due to slow vessel wall degeneration from occupation-related repeated micro trauma. True aneurysms that lack a clear etiology have been termed as ‘spontaneous’ aneurysms.^1^ Here we present a case of a spontaneous true aneurysm of a common digital artery in a 77-year-old female. She underwent successful surgical excision of the lesion and arterial continuity was restored by end-to-end anastomosis of the vessel.

Informed written consent was obtained from the patient before data collection and publication of the case report. The study was done as per the standards of the institutional ethics committee and the Helsinki declaration.

## CASE REPORT

**Figure 1:**
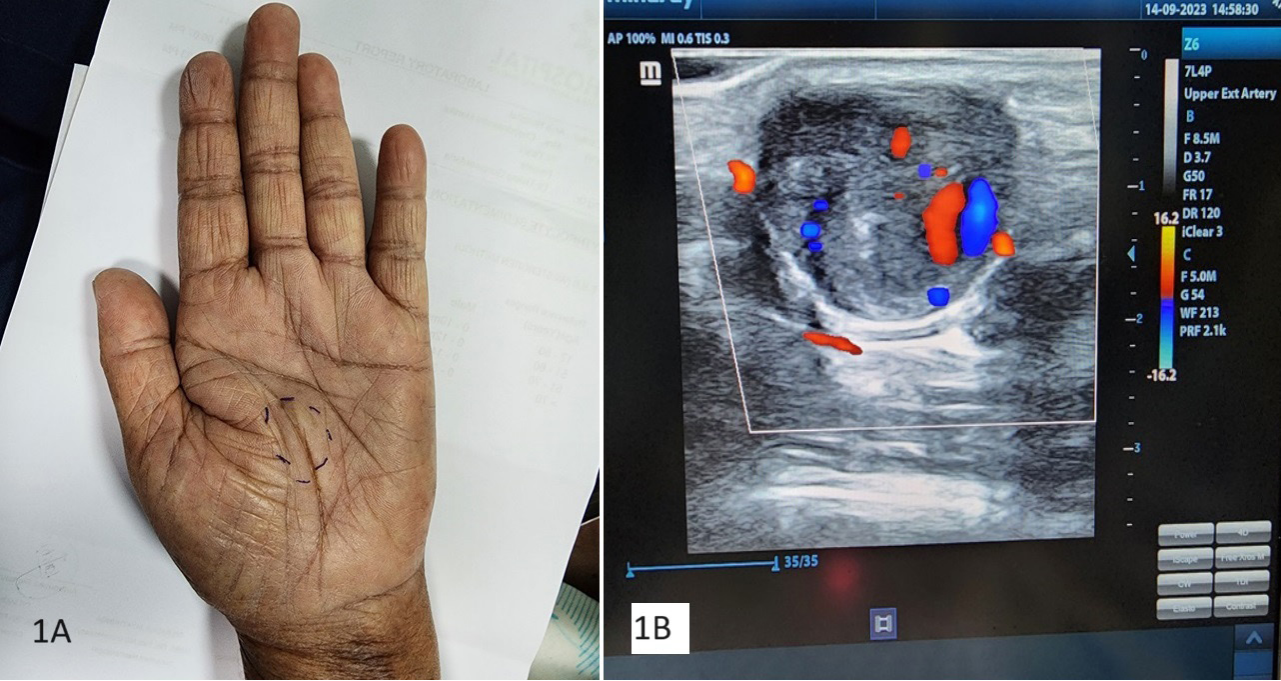
(A) Lump over the palm; (B) Duplex showing the partially thrombosed aneurysm.

A 77-year-old, previously healthy lady was referred to the vascular clinic with a progressively enlarging painful lump over her left palm (Figure 1A). The lump was first noted by the patient approximately 6 weeks back. There was no preceding history of sharp or blunt trauma to the hand and the patient denied the use of mechanical instruments that can cause repetitive soft tissue damage. On examination, the lump was approximately 1 cm x 1 cm in size and pulsatile. The fingers were adequately perfused and both radial and ulnar pulses were well felt at the wrist. Allens’s test confirmed co-dominant radial and ulnar artery perfusion to the hand.

She underwent a duplex ultrasound scan (DUS) of her affected hand which demonstrated a partially thrombosed aneurysm with dimensions of 19 x 16 millimeters (Figure 1B). CT angiogram (CTA) of the upper limb and the hand confirmed these findings. Her blood investigations as well as a 2D echocardiogram were normal. As the lesion was enlarging and symptomatic, surgical repair was offered.

**Figure 2:**
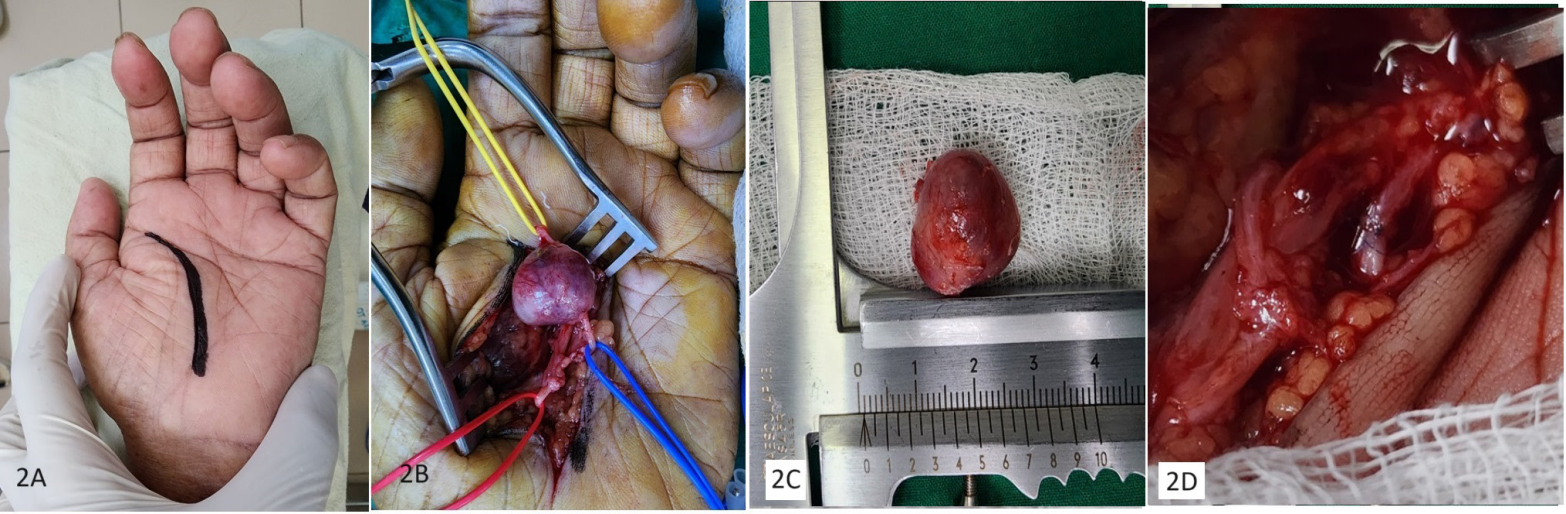
(A) Surgical incision; (B) Dissected aneurysm; (C) Excised specimen; (D) End-to-end reconstruction.

Under general anesthesia, the left hand and forearm were exsanguinated and a tourniquet was inflated at the level of the midarm. The procedure was performed with the aid of 3.5x magnification loupes. An incision was made over a skin crease and the aneurysm was dissected (Figures 2A and 2B). The lesion arose from the common digital artery supplying the left index and middle fingers. After administration of 5000 international units of intravenous unfractionated heparin, Bulldog clamps were applied proximal and distal to the lesion, and the aneurysm was excised (Figure 2C). The continuity of the vessel was restored by end-to-end anastomosis using 7 0 polypropylene interrupted sutures (Figure 2D).

**Figure 3:**
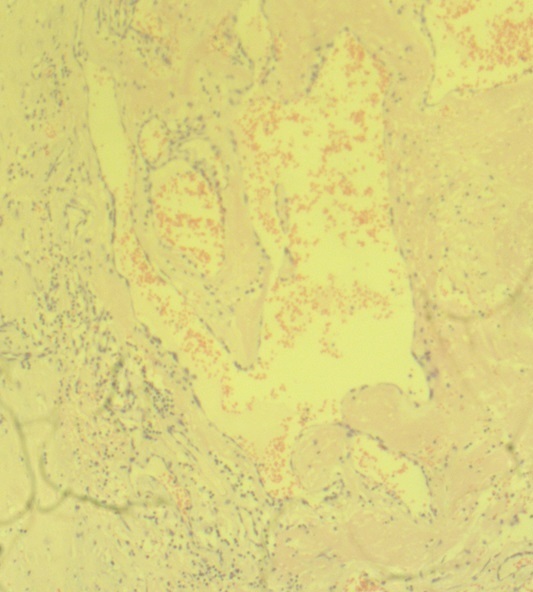
Histology of the excised specimen depicting 3 layers of the arterial wall.

The patient made an uneventful recovery and was discharged on the following day. The histology of the excised lump confirmed it as a true aneurysm (Figure 3). At 6 months follow-up, the patient remained free of recurrence with excellent hand function.

## DISCUSSION

An aneurysm of a hand artery can present as a pulsatile lump or localized pain over the palm. Digital ischemia as a result of thrombosis of the aneurysm and bleeding due to rupture are possible but rare.^2^ Altered sensation over the digits and intolerance to cold exposure can be caused by pressure on the digital nerves.^3^

The distal ulnar artery, palmar arch, and common and proper digital arteries of the hand can be affected by aneurysmal disease. The digital arteries are the least commonly involved of these vessels.^4^ False aneurysms are the most frequently encountered pathological variety.^2^ They are usually secondary to penetrating trauma.^5^ Infection is a rare cause of hand aneurysms. Such mycotic aneurysms can follow septic cardiac emboli or localized hand infections.^2,6^

True hand aneurysms have been associated with repeated micro trauma to the hand, which is commonly related to the patient’s occupation. The relative lack of soft tissue over the hand arteries readily exposes these vessels to compressive forces. This can lead to weakening of the medial layer and fusiform dilatation of the artery.^7^ In some patients with true hand artery aneurysms, no precipitating cause is identified. This was the case in our patient as well. Such lesions have been named ‘spontaneous’ aneurysms.^1^ Although the etiology is not obvious, such cases are likely a consequence of vessel wall degeneration, similar to occupation-related true aneurysms. In children, congenital true aneurysms of the hand have been reported.^3^

When an aneurysm of a hand artery is suspected, the first-line imaging modality to confirm the diagnosis is DUS. Additional information that may aid in operative planning can be obtained by CTA or magnetic resonance angiography. Conventional angiography has been replaced by these non-invasive imaging techniques.^8^

Management strategies for hand aneurysms vary across the literature. Proximal and distal ligation of the artery followed by excision of the lesion is straightforward. However, digital ischemia can be a complication and this can be avoided by ensuring adequate collateral perfusion. A positive Allen’s test will be reassuring as it indicates the hand has a co-dominant blood supply from both radial and ulnar arteries.^8^ In addition, after clamping the vessel feeding the aneurysm, a good Doppler signal over the distal digital arteries can be confirmed intraoperatively. Brisk back bleeding from the distal end of the artery after excision of the aneurysm can also be considered a surrogate marker of adequate collateral perfusion.^9^ Reconstruction after removal of the aneurysm can be done by end-to-end anastomosis or using a venous or arterial interposition graft. Irrespective of the operative technique, recurrence is rare. In our patient, we opted for end-to-end anastomosis after excision of the aneurysm as the vessel ends could be brought together without undue tension. We used interrupted stitches using a fine polypropylene suture to avoid constriction at the suture line.

## CONCLUSIONS

Aneurysms of the hand arteries are rare entities. Operative intervention is preferred over conservative management to alleviate symptoms as well as to prevent complications.^8^ Excision followed by restoration of arterial continuity is the management option of choice.^4^ Despite advances in endovascular management of arterial pathologies, aneurysms of the hand vessels are still managed by open surgery.^8^
